# Macular vascular density changes in different stages of chronic primary angle-closure glaucoma

**DOI:** 10.3389/fmed.2025.1620673

**Published:** 2025-07-24

**Authors:** Juntao Zhang, Qinkang Lu, Huilei Yu, Bowen Liu, Jingwen Yang, Tianyu Wang, Fang Wang

**Affiliations:** ^1^Shanghai Tenth People's Hospital, School of Medicine, Tongji University, Shanghai, China; ^2^The Affiliated People’s Hospital of Ningbo University, Ningbo, China; ^3^Ningbo Key Laboratory of Medical Research on Blinding Eye Diseases, Ningbo Eye Institute, Ningbo Eye Hospital, Wenzhou Medical University, Ningbo, China

**Keywords:** primary angle closure glaucoma, macular vascular density, visual field defects, macular retinal nerve fiber layer, ganglion cell layer, optical coherence tomography angiography

## Abstract

**Objective:**

This study aims to investigate differences in macular vascular density (MVD) between individuals with chronic primary angle-closure glaucoma (CPACG) and healthy controls, as well as to evaluate cross-sectional changes in MVD at various stages of CPACG.

**Method:**

This is a retrospective study based on the epidemiological survey of eye diseases in the local community, including 47 eyes of CPACG subjects (20 eyes at the early stage and 27 eyes at the middle-to-severe stages). All subjects underwent optical coherence tomography angiography (OCTA) imaging to detect MVD, as well as macular retinal nerve fiber layer (RNFL) and ganglion cell layer (GCL) thickness. Linear regression analysis was performed to evaluate other ophthalmic indicators related to vascular density loss.

**Results:**

Compared to the control group, the MVD in CPACG eyes significantly declined by 11.5% in the superficial capillary plexus (*p* = 0.012) and 6.8% in the deep capillary plexus. Single correlation analysis showed that MVD in CPACG eyes was significantly correlated with axial length (r = 0.493, *p* = 0.036), RNFL thickness (r = 0.488, *p* = 0.047), and mean deviation of the visual field (r = −0.546, *p* = 0.010). In addition, multiple regression analysis also suggested that MVD was positively correlated with GCL/RNFL thickness and negatively correlated with the mean deviation of the visual field (*p* = 0.004).

**Conclusion:**

Our study demonstrated that OCTA was a valuable tool for detecting vascular deterioration in CPACG eyes, with a stronger association between MVD and visual field damage. Further research is warranted to explore the potential of MVD as a biomarker for glaucoma progression.

## 1 Introduction

Glaucoma is an optic neuropathy characterized by elevated intraocular pressure (IOP), optic nerve atrophy, and visual field (VF) damage accompanied by the loss of retinal ganglion cells (RGCs). It was reported that by 2040, the number of glaucoma cases among individuals aged 40–80 years will increase from 76 million in 2020 to 111.8 million, representing an increase of approximately 47.1%, mainly attributed to cases in Asia and Africa (1, 2). It is worth noting that in Asia, especially in China, 80% of primary glaucoma cases were angle closure glaucoma (3). Depending on the clinical presentation and severity, glaucoma can be divided into two forms. Acute glaucoma is characterized by the closure of the anterior chamber angle due to a sudden rise in IOP. In contrast, chronic glaucoma involves the closure of the chamber angle or the development of peripheral anterior adhesions due to a gradual increase in IOP (4). Chronic primary angle-closure glaucoma (CPACG) is a distinct type, well-known for its insidious progression and potential to cause significant optic nerve damage.

Studies indicate that PACG is usually associated with obstructed aqueous drainage channels and persistent elevation of intraocular pressure (IOP), which affect retinal circulation and leads to a more extensive loss of vascular density (VD) and a reduction in retinal nerve fiber layer (RNFL) thickness (5). Swept-source OCT or spectral-domain OCT with a wavelength of approximately 1,050 nm could effectively penetrate the retinal pigment epithelium and is widely used for the assessment of RGC and RNFL thickness (6, 7). The segmented mRNFL and ganglion cell layer (GCL) showed moderate sensitivity but high specificity for diagnostics of glaucomatous eyes and was comparable to cpRNFL thickness (6). The optic disc parameters had lower diagnostic accuracy than the RNFLT and GCL parameters (7).

The vascular system and ocular blood flow also play important roles in the pathological process of PACG. High intraocular pressure leads to compression of the soft tissue structure in the sieve plate area, and repeated mechanical compression may also impair vascular regulation function, thereby dynamically affecting microcirculation in the optic disc area and indirectly affecting macular perfusion. The dysregulation of ocular blood flow could trigger neuronal damage in the ischemic phase, leading to thinning of the RNFL and GCL (8). It was reported that disc hemorrhage was a significant negative prognostic factor for non-traumatic glaucoma and might be indicative of progressive damage to RNFL (9). In eyes with primary open-angle glaucoma (POAG), superficial macular vascular density may have diagnostic accuracy comparable to that of pericapillary RNFL and macular GCL thickness (10). Current studies on retinal vascular density in glaucoma mainly focused on the optic nerve head and the peripapillary region (11–13). Weinreb demonstrated that decreased microvascular density was closely associated with the severity of VF damage, even after controlling for the impact of structural loss (11). Yoshimura also reported that in glaucomatous eyes without high myopia, microvascular densities of the optic discs were significantly decreased at locations corresponding to VF defects (*p* = 0.006) (13). The same situation was also proved in the peripapillary location in all subjects with normal-tension glaucoma and POAG (14, 15). An early report on CPACG indicated a significant reduction in vascular blood flow around their capillaries during an 18-month follow-up (16).

However, few studies have focused on the retinal microvasculature in the macular region of PACG eyes. We hypothesized that MVD reduction correlates with glaucoma severity. Therefore, this study aims to investigate whether macular vascular density (VD) correlates with visual field defects or other structural changes in CPACG eyes at various stages.

## 2 Materials and methods

This is a retrospective single-center study based on epidemiological screening data on glaucoma in the Ningbo region from April 2020 to November 2023. This study was designed to identify a standardized effect of size 1.0 with a power of 80% and a risk of a type I error of 5%. Hence, an overall sample size of approximately 32 was required for statistical power. We recruited 47 eyes from 31 CPACG subjects. The inclusion criteria for CPACG eyes were listed as follows (17, 18): (1) the closure angle should be confirmed, (2) presence of glaucoma-related optic neuropathy and VF damage, (3) long-term elevated IOP (>21 mmHg for ≥6 months), and (4) none of the symptoms or signs of acute attacks. The exclusion criteria included the following: (1) history of intraocular surgery (excluding cataract surgery without complications); (2) history of neurodegenerative diseases, including head trauma, stroke, Alzheimer’s disease, and Parkinson’s disease; (3) diabetes or other causes of retinal lesions (cup-to-disc ratio abnormalities or RNFL damage); (4) patients with severe cataracts (LOCS III grade ≥4) or high myopia, (5) history of acute angle-closure glaucoma attacks; and (6) history of any kind of glaucoma treatment.

According to the degree of VF damage, glaucoma staging could be divided into mild (VFMD ≥ − 6 dB) or moderate to late stage (VFMD < −6 dB, moderate defined as −6 to −12 dB, advanced defined as <−12 dB) (19). In this study, 28 healthy participants were included with mild to moderate cataracts but no other eye diseases. One eye was randomly selected from the normal controls. The research program was conducted in accordance with the Declaration of Helsinki and approved by the Institutional Review Board of the affiliated hospital of Ningbo University. Informed consent was obtained from all participants or their legal guardians.

After enrollment, information from all participants was collected, such as name, gender, date of birth, and chronic medical history. Comprehensive ophthalmic examinations were conducted, including vision, IOP by non-contact tonometer (KOWA, KT-800, Japan), cornea by slit lamp biomicroscopy (SLM-5E, Kanghua Ruiming, Chongqing, China), fundus examination by laser scanning ophthalmoscope (Daytona (P200T), Oubao, UK), and axial length by IOL Master 700. Visual field defect was also tested by a fully automatic visual field analyzer (Humphrey840, ZEISS, Germany), with a spot size of 2 mm, holding time of 600 ms, and background light intensity of 4 asb.

### 2.1 Optical coherence tomography angiography

**Figure 1:**
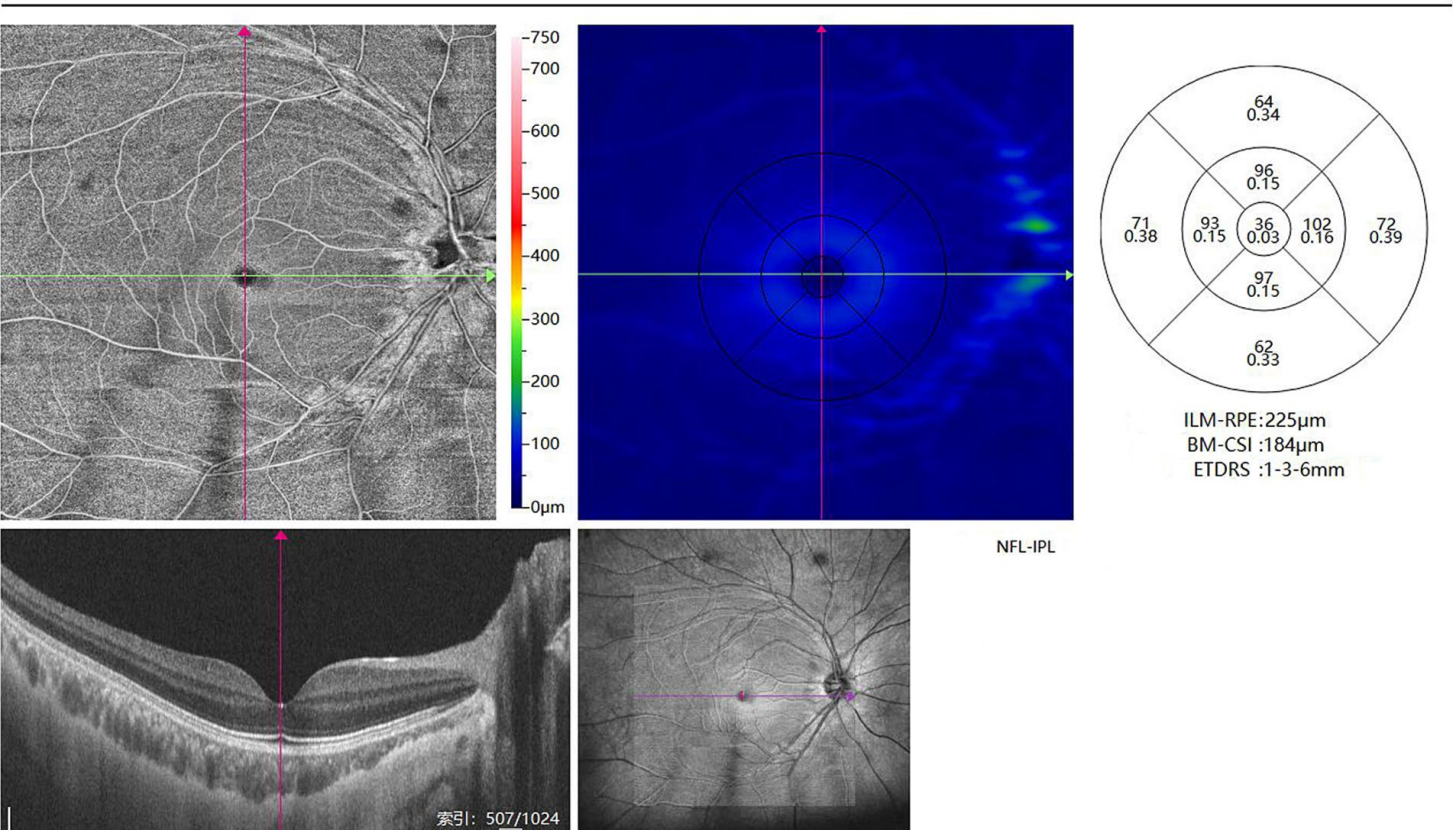
OCTA could detect the thickness of different layers of the retina. In the coronal direction, the macular region was scanned in the region of 6 mm × 6 mm and 3 mm × 3 mm centered on the fovea. RNFL and GCL thickness were defined as the mean value in the entire annular area of the inner ring (3 mm × 3 mm).

Retinal angiography was performed by experienced ophthalmologists using the newly developed ultrawide-field optical coherence tomography angiography (UWF-OCTA) (BM-400 K, Tupai Medical, Beijing, China) with a laser light wavelength of 1,060 nm (24 mm × 20 mm scan area, A/B-scan count of 1,536/1,280, axial resolution 3.8 μm, lateral resolution 10 μm). Structural indicators were analyzed using OCTFunduslmage3 software (1.3.3.1 version). The defined region was centered on the fovea with motion correction. Macular vascular density (MVD) was quantified by measuring the percentage of blood flow perfusion, and large vessels were excluded from the calculation (20). MVD was obtained by scanning a region of 3 mm × 3 mm centered on the fovea. The split-spectrum amplitude-decorrelation angiography algorithm was applied to automatically segment the superficial vascular complex, the deep vascular complex, the choriocapillaris layer, and the choroidal vessel layer. All these layers could be quantified on vessel density with relatively high inter-observer reproducibility (0.66–0.94). All the primary data were exported by the built-in software of the SS-OCT and SS-OCTA platforms. The thickness of RNFL and GCL could also be automatically quantified (Figure 1). The quality of each OCT angiography image was evaluated, and low-quality scans with a signal strength index <60 were excluded.

### 2.2 Statistical analysis

The normality of quantitative data was evaluated using the Kolmogorov–Smirnov normality test. The Kolmogorov–Smirnov normality test was conducted by comparing the cumulative distribution function of the sample data with the cumulative distribution function of a normal distribution. A *p*-value of greater than 0.05 was considered to indicate normality. All continuous variables were expressed as mean ± standard deviation. All categorical variables were expressed in frequency (percentage). Analysis of variance (ANOVA) was used to evaluate differences among the controls and CPACG subgroups; post-analysis Bonferroni test was used to compare the differences among groups. The χ^2^ test was used to evaluate the differences among groups by age and sex distributions. Pearson’s correlation analysis was used to study the relationship between MVD and traditional functional and structural indicators. Subsequently, the stochastic expectation maximization algorithm was applied to deal with bilateral data, and multiple linear regression analysis was used to evaluate the association between MVD and axial length, mean deviation of visual field, GCL, and RNFL. All statistical analyses were conducted using SPSS software package version 20.0, with a *p*-value of <0.05 indicating statistically significant differences.

## 3 Results

### 3.1 Comparison of general clinical data

This study recruited 28 healthy eyes and 47 eyes from 31 CPACG subjects (20 mild eyes and 27 moderate-to-advanced eyes). Table 1 displays general data on age, gender, IOP, axial length, mean deviation of visual field (VFMD), GCL, RNFL thickness, and C/D area ratio. Except for IOP, age, and gender, there were significant differences in axial length, visual field damage, C/D area ratio, GCL, and RNFL thickness between CPACG subjects and normal controls (*p* < 0.05). For CPACG eyes at different stages, significant changes in retinal structure were also shown in moderate-to-advanced eyes with the most severe visual field damage (*p* < 0.001), increased C/D area ratio, and thinner GCL and RNFL thickness (*p* < 0.001).

**Table 1:** Demographic data and clinical characteristics of all CPACG patients.

Parameter	Controls (A)	Mild subgroup (B)	Moderate-to-advanced subgroup (C)	*p*-value
Eyes (*n*)	28	20	27	
Age (years)	71.6 ± 10.3	69.5 ± 13.4	72.2 ± 9.8	Overall: 0.76
Sex (male/female)	71/81	48/56	52/69	Overall: 0.34
IOP (mm Hg)	14.78 ± 5.62	15.54 ± 7.33	16.32 ± 6.74	Overall: 0.49
Axial length (mm)	24.1 ± 2.3	22.4 ± 2.1	22.1 ± 1.9	Overall: 0.040 A–B: 0.035 A–C: 0.022 B–C: 0.86
VFMD (dB)	2.57 ± 2.36	5.32 ± 3.54	12.46 ± 4.26	Overall: <0.001 A–B: 0.017 A–C: <0.001 B–C: <0.001
C/D ratio	0.41 ± 0.17	0.50 ± 0.22	0.63 ± 0.26	Overall: 0.023 A–B: 0.064 A–C: 0.012 B–C: 0.047
RNFL thickness (μm)	103.3 ± 18.7	92.6 ± 18.1	71.5 ± 17.4	Overall: <0.001 A–B: 0.009 A–C: <0.001 B–C: <0.001
GCL thickness (μm)	103.8 ± 21.4	94.9 ± 16.7	77.6 ± 12.8	Overall: <0.001 A–B: 0.016 A–C: <0.001 B–C: <0.001

IOP, intra-ocular pressure; C/D ratio, vertical-based cup-to-disc ratio; RNFL, retinal nerve fiber layer; GCL, ganglion cell layer; VFMD, mean deviation of visual field.

### 3.2 Determination of MVD in multiple layers of the macular region

**Figure 2:**
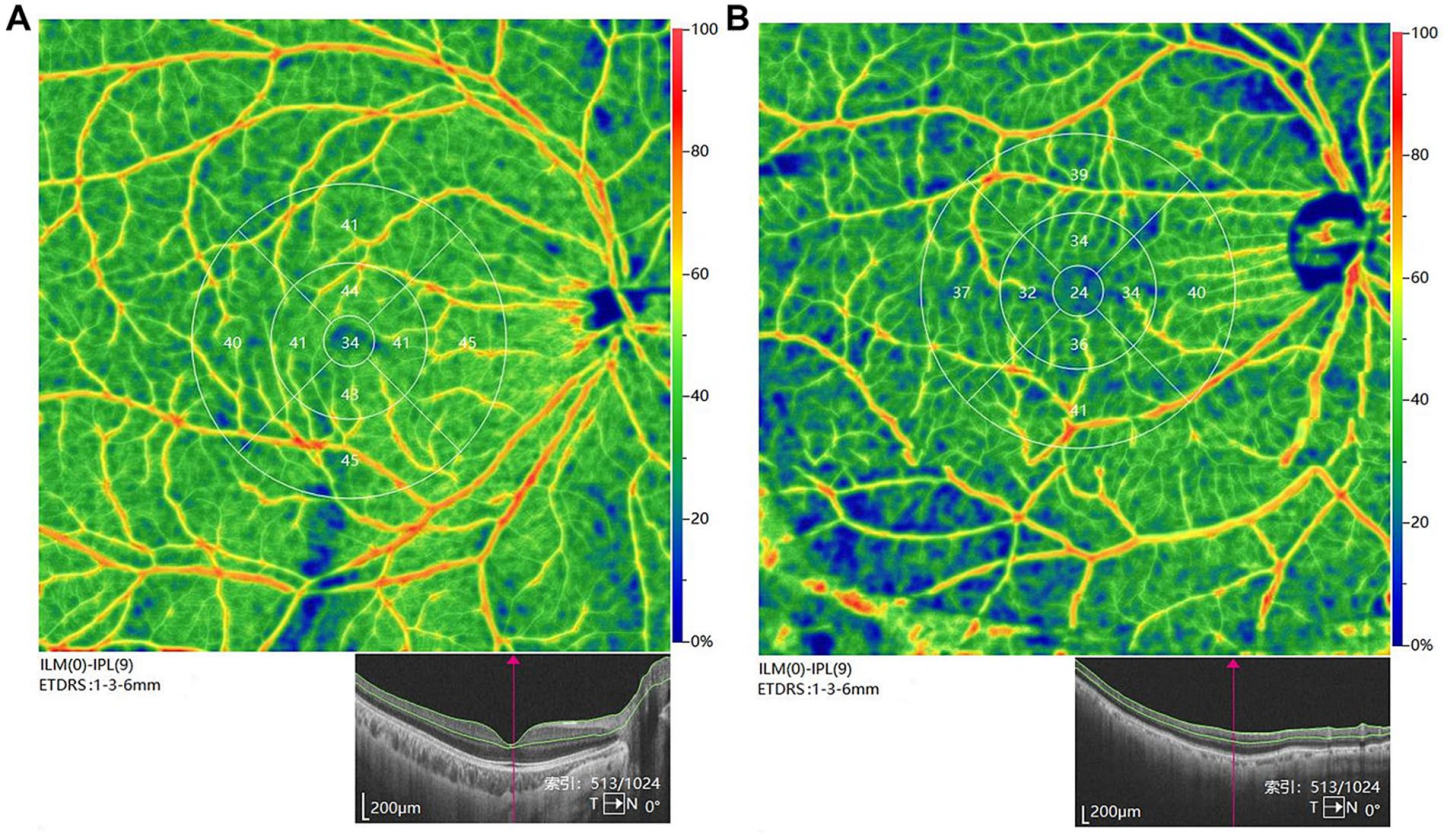
Representative image of vascular perfusion density in the superficial capillary plexus (ILM(0)-IPL(9)) in the region of 6 mm × 6 mm and 3 mm × 3 mm centered on the fovea [**(A)** normal control; **(B)** CPACG eye]. Fovea was defined as an area with an inner diameter of 1 mm centered on the fovea centralis. Parafovea was defined as the entire annular area of the inner ring (3 mm × 3 mm), which was also divided into four blocks: temporal, superior, nasal, and inferior.

According to the coronal images of the OCTA scan, the microvascular network in the normal eyes was denser than that in the CPACG eyes, since it became sparse in the CPACG eyes and showed varying degrees of macular atrophy and expansion of capillary spaces (Figure 2). In addition, the pseudo-color images of blood vessels showed that the blood vessel density was lower in CPACG eyes.

OCTA can measure vascular density across multiple layers of the macular region, primarily including the superficial capillary plexus, deep capillary plexus, and choriocapillaris layers. Compared with the normal controls, the differences of MVD in CPACG eyes were mainly reflected in the superficial capillary plexus and deep capillary plexus (Table 2). Compared to normal controls, MVD in CPACG eyes declined by 11.5% in the superficial capillary plexus and by 6.8% in the deep capillary plexus. Among CPACG eyes at different stages, a significant decrease in MVD was observed only in the superficial capillary plexus of moderate-to-advanced eyes (45.38 ± 3.14% in controls vs. 38.61 ± 2.89% in advanced CPACG), compared to early-stage eyes (*p* = 0.012). No significant differences in MVD were found in other capillary plexuses.

**Table 2:** Measurement of MVD in the CPACG subgroups and the control group.

Parameter	Controls (A)	Mild subgroup (B)	Moderate-to-advanced subgroup (C)	*p*-value
Superficial (%)	45.38 ± 3.14	41.84 ± 3.11	38.61 ± 2.89	Overall: <0.001 A–B: 0.003 A–C: <0.001 B–C: **0.012**
Deep (%)	48.56 ± 3.05	46.17 ± 3.49	44.79 ± 3.28	Overall: <0.001 A–B: 0.031 A–C: 0.004 B–C: 0.11
Choroid capillary (%)	62.79 ± 2.83	62.57 ± 4.69	62.14 ± 4.02	Overall: 0.63

*p*-values are presented for the overall one-way ANOVA, followed by pairwise comparisons with the Bonferroni correction. The bold values represented statistically significant.

In the superficial capillary plexus, MVD changes were most pronounced. The parafovea region was divided into four parts: temporal, superior, nasal, and inferior. The results showed that the MVD changes in parafovea were more obvious than those in the fovea area (overall: *p* < 0.001), and could clearly distinguish CPACG eyes at different stages (Table 3). Compared to early eyes, the MVD in moderate-to-advanced eyes decreased by 5.6% in the parafovea region (*p* = 0.042). The MVD changes in temporal and inferior areas were more remarkable between early eyes and moderate-to-advanced eyes, with a reduction of 7.5% (*p* = 0.006) and 4.8% (*p* = 0.035), respectively.

**Table 3:** MVD changes in different regions of the superficial capillary plexus.

Parameter	Controls (A)	Mild subgroup (B)	Moderate-to-advanced subgroup (C)	*p*-value
Fovea (%)	26.51 ± 5.02	24.65 ± 4.13	24.07 ± 3.96	Overall: 0.032 A–B: 0.015 A–C: <0.001 B–C: 0.86
Parafovea (%)	53.12 ± 3.73	48.83 ± 4.23	46.12 ± 3.91	Overall: <0.001 A–B: <0.001 A–C: <0.001 **B–C: 0.042**
Temporal (%)	52.65 ± 3.91	48.21 ± 3.79	44.62 ± 5.01	Overall: <0.001 A–B: <0.001 A–C: <0.001 **B–C: 0.006**
Superior (%)	52.71 ± 4.24	48.09 ± 4.41	47.75 ± 5.03	Overall: 0.012 A–B: <0.001 A–C: <0.001 B–C: 0.74
Nasal (%)	52.80 ± 3.91	48.39 ± 4.23	47.52 ± 4.88	Overall: 0.014 A–B: <0.001 A–C: <0.001 B–C: 0.43
Inferior (%)	54.85 ± 4.04	48.12 ± 4.87	45.81 ± 6.03	Overall: <0.001 A–B: <0.001 A–C: <0.001 **B–C: 0.035**

The bold values represented statistically significant.

### 3.3 The correlation between macular blood flow density and structure

Pearson’s correlation analysis showed no relationship between MVD and IOP, C/D ratio, or age (Table 4). There was a negative correlation between vascular density and mean deviation of the visual field (*β* = −0.90, *p* = 0.010), and a positive correlation between vascular density and GCL thickness (*β* = 0.35, *p* = 0.019), RNFL thickness (*β* = 0.46, *p* = 0.047), and axial length (*β* = 0.45, *p* = 0.036).

**Table 4:** Correlations between the superficial MVD and other glaucoma parameters in all CPACG.

Variables	MVD		
	*r*	*p*-value	Standard β
Age	−0.331	0.158	−0.36
Cup-to-disc ratio	−0.374	0.093	−0.53
Axial length	0.493	**0.036**	0.45
GCL thickness	0.516	**0.019**	0.35
RNFL thickness	0.488	**0.047**	0.46
IOP	−0.401	0.070	−0.50
Mean deviation of the visual field	−0.546	**0.010**	−0.90

The bold values represented statistically significant.

Multiple linear regression analysis was performed on all participants to identify factors independently associated with MVD (Table 5). The results showed a negative correlation between vascular density and mean deviation of the visual field [OR = −0.21 (−0.17, −0.26), *p* = 0.004]; and a positive correlation between vascular density and GCL thickness [OR = 0.18 (0.13, 0.23), *p* = 0.009]. In the multiple regression analysis, variables such as axial length, GCL/RNFL thickness, and VFMD maintained independent correlation. Additionally, insufficient sample size or statistical power may have affected the results of the multivariate regression analyses to some extent.

**Table 5:** Multiple linear regression analysis of the superficial MVD in all CPACG.

Variables	MVD		
	OR	95% CI	*p*-value
Axial length	0.11	0.08, 0.19	**0.014**
GCL thickness	0.18	0.13, 0.23	**0.009**
RNFL thickness	0.15	0.10, 0.19	**0.026**
Mean deviation of the visual field	−0.21	−0.17, −0.26	**0.004**

The bold values represented statistically significant.

## 4 Discussion

Previous studies indicate that the macular region plays a crucial role in visual impairment in glaucomatous eyes. Therefore, the macula is recommended as an important site for evaluating glaucoma (10). Retinal ganglion cells are densely located within the macula (21). Through digital imaging, scientists found that the diameters of retinal arteries and veins in PACG eyes were restricted (22). It was inferred that mechanical compression might temporarily block blood flow through the eyes. If IOP can be effectively controlled, the microcirculation system might reopen to a certain degree. It has been reported that there are potential relationships among MVD, best corrected visual acuity, and the macular ganglion cell complex, indicating a correlation between a sharp decline in MVD and severe visual impairment (23). Dr. Kim et al. found that the thickness of the macular GCL and cpRNFL was prominent in the differential diagnosis of early, middle, and late glaucoma. Loss of RGCs can result in a reduction of macular thickness, which is associated with RNFL thinning and VF defects (24). Vascular blood flow directly supports the metabolic needs of RGCs, and changes in their density may better reflect the blood supply and damage to these cells, while visual field defects are directly related to the functional status of RGCs. However, high IOP is only one risk factor for injury, and its impact may indirectly lead to injury through multiple mechanisms. Its correlation with functional impairment may not be as strong as direct blood flow measurement (25, 26). Another study also suggested that in differentiated POAG and unaffected eyes, the diagnostic accuracy of MVD was higher than others, such as cpRNFL and GCL thickness (27). The retinal and choroidal VD in glaucoma was significantly reduced, especially in the early stages of the disease, which meant that VD changes might occur earlier and be more sensitive to changes in retinal structure (28). It indicated that MVD was of great significance in monitoring the progression, severity, and prognosis of glaucoma. This hypothesis needs to be verified through further clinical studies in a larger sample size.

It was first reported that across the primary angle closure disease spectrum, only PACG showed significant microvascular reduction, which was positively correlated with VFMD (29). Previous studies mainly focused on the impairment of macular microcirculation in acute PACG patients (30, 31). For the innovation of this study, we conducted a systematic analysis of MVD changes in different regions of the superficial capillary plexus in CPACG, and multiple linear regression analysis was performed to identify correlations between the superficial MVD and other glaucoma parameters. More importantly, all CPACG participants recruited were from Eastern coastal regions of China, which had rarely been reported in the epidemiological studies about glaucoma (32, 33). Our results indicated that the significant vascular deterioration of the macular superficial capillary plexus in CPACG eyes could be differentiated by OCTA. For the advanced CPACG eyes, the disappearance rate of MVD was markedly faster with a lower MVD baseline. A previous study reported a significantly lower MVD in advanced POAG eyes (34). Our results showed that compared to the normal controls, the differences of MVD in CPACG eyes were mainly reflected in the superficial capillary plexus and deep capillary plexus. For moderate-to-advanced eyes, the significant MVD decrease was shown only in the superficial capillary plexus compared to early eyes. This situation was different from other eye diseases, such as pathological myopia. The decrease in blood flow density in the deep capillary of patients with pathological myopia was more pronounced than in the superficial capillary, but the changes in each region were relatively uniform (35).

In addition, we also compared the VD value in different regions of the macula’s superficial capillary plexus. Compared with early CPACG eyes, the MVD decrease in moderate-to-advanced eyes was more significant in the temporal and inferior regions. It was consistent with previous studies suggesting that the greatest decline of VD in POAG eyes was predominantly in the macular region, especially in the infratemporal part (36, 37). It was suggested that the loss of retinal ganglion cells and their axons was selective, with the worst damage in the inferior and temporal quadrants, and that the microvascular damage in the macula started from the inferior-posterior and temporal-posterior quadrants, both of which coincided with the structural map of the axons and the visual field defects (37, 38).

Our results indicated that the deterioration of MVD over time in glaucomatous eyes might not be related to IOP. Macular microvascular injury in glaucoma might not only be a secondary result of elevated IOP but also an important cause of the development and progression of glaucoma. It was speculated that IOP did not affect the diagnostic efficacy of MVD in glaucomatous eyes, which was consistent with the relevant report (39). Besides, literature reported that a rapid decline in visual field damage was significantly correlated with age, hypertension, blood flow density, and other factors (40). Our results were different and showed that MVD changes were not related to age, but closely associated with axial length, GCL/RNFL thickness, and visual field damage.

In the normal population, a longer axial length (myopia) is usually accompanied by deeper intraocular structures, which theoretically provides a better microvascular spatial distribution. The axial length of CPACG patients is significantly shorter than that of the normal population, leading to a decrease in MVD and exacerbating vascular damage. Some clinical studies supported this viewpoint (41, 42), while another study suggested that the effect of axial length on retinal microvascular density depended on the measurement region (43). In the inner loop, VD significantly increased with increasing axial length, while in the outer ring, VD decreased as axial length increased. Linear regression analysis showed a positive correlation between inner ring VD and AL, and a negative correlation between outer ring VD and AL. Additionally, similar to findings in POAG studies, a positive correlation was found between MVD and visual field defects in CPACG patients (23, 34, 44). Unlike POAG, the decrease in vascular density in PACG may be independent of thinning of the nerve fiber layer (45), and macular vascular density is more predictive of visual field progression than optic disc vascular density (R^2^ = 12.5% vs. 3.3%) (40). Overall, MVD is considered a useful alternative indicator for analyzing the progression of glaucoma.

There were several limitations to this study. First, as a cross-sectional study, it was impossible to determine a causal relationship between vascular flow density and structural changes during glaucoma progression. The data from cross-sectional studies were collected at the same time point, making it difficult to determine causal relationships between variables and only providing associations, which may result in selection bias and recall bias. Additionally, the relatively small sample size may limit the statistical power of the study, potentially affecting the accuracy and reliability of the results. Future studies with larger sample sizes are needed to further validate our findings. Further longitudinal studies are needed to assess the ability of OCTA to predict glaucoma progression. Opacity of ocular media, such as cataracts, corneal edema, and vitreous hemorrhage, may interfere with image layering, leading to the misjudgment of vascular density or position. Opacity of ocular media is one of the common causes of OCTA artifacts, which may lead to bias and other issues, thereby affecting diagnostic accuracy. Finally, considering the multifactorial nature of MVD parameters, supplementary qualitative and quantitative macular vascular metrics should be considered in further studies to strengthen the study’s robustness.

## 5 Conclusion

This study indicated that OCTA could detect vascular deterioration in CPACG patients at different stages. MVD loss was more severe in advanced CPACG eyes. There was a close correlation between MVD, visual field damage, axial length, and GCL/RNFL thickness. Additionally, MVD is most closely associated with visual field damage.

## Data Availability

The raw data supporting the conclusions of this article will be made available by the authors, without undue reservation.
